# Sharing data, sharing methods, sharing science.

**DOI:** 10.1016/j.mex.2021.101607

**Published:** 2021-12-14

**Authors:** Sergio Pantano

**Affiliations:** Institut Pasteur de Montevideo, Montevideo, Uruguay

## Introduction

Research and discovery in the most disparate areas of knowledge are constantly being pushed by continuous technological revolutions. The permanent waves of advances changed how scientific work is planned, produced, and communicated within the scientific community and made available to the general public. The "big-data revolution" impacted practically all scientific disciplines, increasingly imposing data-driven methodologies [1]. A significant outcome of this process is a shift towards open data initiatives (Budapest OS initiative, Plan S, EOSC, etc.). These initiatives have contributed to greater availability and accessibility of publicly funded scientific research, accompanied by the creation of freely available data repositories for large volumes of information.

Soon after these initiatives started, the necessity to improve data organization, and storage efficiency and effectiveness became evident. As a result, FAIR (findable, accessible, interoperable, reusable) guiding principles for scientific data management and stewardship were outlined [2,3].

The large-scale data accessibility powered a plethora of new analytical tools such as those commonly bundled under the generic denomination of artificial intelligence [4], [5], [6], [7], [8].

Obviously, the capacity to sort, analyze and integrate this overwhelming amount of information is extremely challenging and demands particular computational skills. Indeed, a minimal computational background is no longer an asset in science; it has become a must. However, achieving simultaneously a comprehensive knowledge of the fundamentals of a particular scientific discipline *and* highly specific computational skills is unlikely to become a standard. Modern science is carried out by interdisciplinary and complementary working teams that, at their time, thrive from knowledge achieved by larger scientific communities. These organizational dynamics require a second level of openness, the sharing of methods and computational analysis tools. Sharing software for data analysis saves the precious time needed for coding, testing, debugging, and documenting, obviating the need for highly specialized computational skills.

Just like the raw data, the nature of the software needed to process it may be of different nature and produced in many formats, languages, and operative systems. It may sometimes be a single software package or pipelines (namely, an array of processing elements organized so that the output of the previous step becomes the input of the next). Clearly, the interoperability of the software and well-documented instructions for compiling and running the software in suitable hardware architectures are mandatory in all these cases.

MethodsX aims to boost science openness at various levels by improving reproducibility, making methods, protocols, and the associated research more discoverable, opening doors for collaboration, and enhancing the research cycle. In line with these aims, MethodsX recently published a number of software packages and pipelines for data analysis to aid research at different levels and in various disciplines. The following paragraphs will briefly overview some specific examples and how they may help within and beyond the scientific community.

## Software utilities for molecular simulations

The increasing computer power and consistently faster algorithms have taken molecular simulations to the level of computational microscopes that enable the study of molecular systems at an unprecedented level [9]. However, applications in material sciences and biology are still critically dependent on stating conditions, e.g., the initial 3D coordinates of the atoms that compose the molecular systems. Recently a couple of exciting applications published in our journal addressed this issue. The software *Nanosculpt* [10] provides a simple method to generate atomic coordinates of complex objects incorporating topological information taken directly from experimental data (as Cryoelectron microscopy/tomography) or user-created arbitrary shapes. In another publication, Gupta et al. [11] analyzed current problems associated with the initial thermalization of the molecular systems and provide an optimized procedure in which nanocrystalline materials are thermalized by coupling specific regions of the simulation box to separated thermostats at different target temperatures.

Although tested only for nanomaterials, both methods also harbor great potential for biological applications.

## Bioinformatics and molecular biology

The wide availability of different sequencing techniques made advanced bioinformatic methods a pressing necessity to get the most out of the data.

The reconstruction of gene phylogenies is based on large sequence alignments. However, homologous sequences are often difficult to align unambiguously, or alignments may contain insufficient information to accurately model gene evolution, leading to incorrect gene trees and erroneous predictions of events of duplications and losses [12]. A workaround for this problem is the construction of longer "supergenes" that comprise sets of loci with putatively similar genealogical stories. However, validating the concatenation of several genes in one single supertree remains a difficult task. Adams and Castoe recently published a model-based protocol for assessing the accuracy of supergenes construction based on phylogenetic congruency that may validate supergene hypotheses [13].

The combination of chromatin immunoprecipitation (ChIP) with sequencing (ChIP-Seq) constitutes a powerful method for identifying genome-wide DNA binding sites. The technique is conceptually simple: DNA-bound proteins are co-immunoprecipitated, purified, and sequenced. However, identifying contiguous specific sequences that act as super-enhancers by binding different transcription factors is computationally highly demanding. Orlova et al. devised a cost-efficient strategy based on distributed computing in virtual machine cloud environments [14]. This method is particularly well suited for research centers with modest access to computational resources and, in principle, independent of the operative system used, making it robust and interoperable.

Another example of cost-efficient bioinformatic methodology but applied to pharmacology is provided by the work of Zidan et al. [15]. They introduced a computational pipeline for pharmacovigilance/pharmacogenomics named PHARMIP. It combines chemical structure and database-reported information about specific drug candidates to anticipate the genetic factors underlying the drug-reported adverse reactions. The implementation is available with a user-friendly interface that facilitates the use by non-experts and can provide valuable hints about genetic risk factors or propensities for specific drug candidates.

## Imaging

The use of fluorescent probes for imaging has revolutionized our understanding of countless biological processes, as it provides non-invasive means to interrogate living organisms in real-time. Nevertheless, experiments may generate a considerable amount of data that need to be processed in different manners. A nice pipeline that offers a semiautomated treatment of time-lapse fluorescence microscopy images, quantification of individual cell signals, and a statistical analysis of the data was recently provided by Lévy et al. [16]. The pipeline combines routines in the popular platforms FIJI, R, and MATLAB packages, automatizing the analysis of a large number of samples and increasing its statistical robustness.

Fluorescent microscopy also grants the opportunity to follow single-molecule localization, unraveling the subcellular organization of different molecular species. Hoboth et al. presented a Single Molecule Localization Microscopy (SMLM) approach to follow the nuclear localization of phosphatidylinositol 4,5- bisphosphate using indirect immunofluorescence labeling [17]. They developed a tool within the free software ImageJ2, which is orthogonal but highly complementary to the more traditional biochemical and lipidomic analyses.

Horzum et al. devised another ImageJ application to process immunofluorescence images of vinculin, a well-characterized marker of cellular focal adhesion [18]. Their "Step-by-step quantitative analysis of focal adhesions" provided a practical approach to quantify a variety of fluorescent images improving the signal-to-noise relationship in systems with high background.

Finally, a pipeline incorporating a macro in the Fiji environment and R scripts to measure the intensity of intracellular staining was reported by Zonderland et al. [19]. This pipeline allows for simple measuring of staining intensities straightforwardly and independently of the cellular shapes or sizes.

## Miscellaneous applications

The packages, pipelines, and methods outlined in the previous paragraphs constitute examples related to some specific areas of research. However, just to illustrate the wide range covered by our journal, we mention a few examples of software with diverse areas of application. The work by Cornish et al. [20] presents a script in phyton language to interrogate the PubMed database for different variations and permutations of eponyms and names given to genes, proteins, and chemical compounds. This simple method enables a fast and unambiguous search, sorting, and characterizing scientific citations by all PubMed's data fields.

A completely different example is provided by the contribution by Arenas-Castro and Gonçalves [21]. They offered a model-assisted method to forecast the suitability of crop production in different terrains as a function of climate change predictions. This machine learning approach written in the R package is freely available on GitHub and may provide helpful information for agricultural sciences and decision-makers about long-term farming politics.

The last example regards a machine learning approach for accurately estimating the mortality produced by pandemic or epidemic events. This is done by calculating the excess mortality from retrospective data using linear regression approaches [22]. Although applied to estimate mortality caused by Covid-19 in Italy in 2020, this approach can be generally applied to obtain valuable epidemiologic insights, keeping updated information about the progress and effect of successive waves of contagions and the effectiveness of containment or vaccination measures.

## Conclusions and outlook

As evident from the articles bundled in this editorial piece, methods-sharing initiatives from scientists for scientists are fervent and extended to different areas. However, significant challenges remain. They are associated with the software's interoperability and robustness to different environments and operative systems, the standardization of data formats used as input and generated as output of the analyzes. Using "software containers" (e.g., software packages that provide applications, dependencies, system libraries, settings, and other binaries, and all the configuration files needed to run) constitutes an effective workaround for the first problem. However, standardization remains a central problem in many areas and still needs to be addressed as a community effort.

Sharing analysis software and computational protocols may create positive feedback loops to progressively overcome these limitations and make high-quality methods more broadly accessible.
